# Phosphomannomutase 2-congenital disorder of glycosylation: exploring the role of N-glycosylation on the endocrine axes

**DOI:** 10.3389/fendo.2025.1594118

**Published:** 2025-07-23

**Authors:** Giulia Del Medico, Lorenzo Ferri, Elena Procopio, Giosuè Annibalini, Elena Barbieri, Rita Barone, Renzo Guerrini, Amelia Morrone, Stefano Stagi

**Affiliations:** ^1^ Health Sciences Department, University of Florence, Florence, Italy; ^2^ Department of Neuroscience and Medical Genetics, Meyer Children’s Hospital Istituto di Ricovero e Cura a Carattere Scientifico (IRCCS), Florence, Italy; ^3^ Metabolic and Muscular Unit, Meyer Children’s Hospital Istituto di Ricovero e Cura a Carattere Scientifico (IRCCS), Florence, Italy; ^4^ Department of Biomolecular Sciences, University of Urbino Carlo Bo, Urbino, Italy; ^5^ IIM, Interuniversity Institute of Myology, Perugia, Italy; ^6^ Child Neurology and Psychiatry Unit, Department of Clinical and Experimental Medicine, University of Catania, Catania, Italy; ^7^ Research Unit of Rare Diseases and Neurodevelopmental Disorders, Oasi Research Institute-Istituto di Ricovero e Cura a Carattere Scientifico (IRCCS), Troina, Italy; ^8^ Department of NEUROFARBA: Neurosciences, Psychology, Pharmacology and Child Health, University of Florence, Florence, Italy; ^9^ Auxoendocrinology Unit, Meyer Children’s Hospital Istituto di Ricovero e Cura a Carattere Scientifico (IRCCS), Florence, Italy

**Keywords:** congenital disorder of glycosylation, endocrine dysfunction, hypoglycosylation, hypergonadotropic hypogonadism, N-glycosylation

## Abstract

Congenital disorders of glycosylation (CDG) are a heterogeneous group of inborn errors of metabolism caused by impaired protein glycosylation. Among these, PMM2-CDG, caused by defective phosphomannomutase 2 activity and affecting protein N-glycosylation, is the most prevalent. As glycoproteins are involved in almost every physiological process, the clinical manifestations in PMM2-CDG are diverse and multisystemic. In the endocrine system, glycoproteins are present in every axis, acting as hormones, prohormones, receptors, enzymes, and transport proteins. Hypoglycosylation can alter hormonal function on multiple levels. As a result, endocrinopathies are frequently part of the clinical spectrum of PMM2-CDG, particularly hypergonadotrophic hypogonadism and pubertal abnormalities in female patients. Symptoms of endocrine involvement, especially hyperinsulinemic hypoglycemia and failure to thrive during infancy, can be the presenting sign of the disease. The clinical spectrum of PMM2-CDG endocrinopathy is variable; for example, thyroid involvement can range from isolated transitory hyperthyrotropinemia to clinical hypothyroidism. Some endocrine abnormalities, such as adrenal insufficiency, are uncommon and probably underdiagnosed in PMM2-CDG. The new insights into the role of N-glycosylation on the endocrine system over the past twenty years have deepened our understanding of this complex disorder and should enable us to improve and personalize the clinical management of these patients.

## Introduction

Phosphomannomutase 2 (PMM2)-congenital disorder of glycosylation (CDG) (previously CDG-Ia; OMIM # 212065) is the most common disorder of protein N-glycosylation in humans, affecting over 1000 patients worldwide (1). It results from biallelic pathogenic variants in the *PMM2* gene, encoding the PMM2 enzyme (OMIM * 601785), which catalyzes the conversion of mannose-6-phosphate to mannose-1-phosphate. This step is required for the synthesis of guanosine diphosphate mannose (GDP-Man) and dolichol-phosphate mannose (Dol-Man). Both are key substrates for the assembly of lipid-linked oligosaccharides which are then added to the asparagine residues of the polypeptide chain. Impairment of PMM2 disrupts the early steps of the glycan assembly chain, resulting in the hypoglycosylation of N-linked glycoproteins which compromises protein folding, stability, translocation and overall functionality (1–3).

Biochemical screening for altered N-glycosylation is based on the identification of abnormal glycoforms of serum transferrin by isoelectric focusing (transferrin isoelectric focusing or TIEF) or mass spectrometry. While these methods reveal abnormal glycosylation they do not identify the specific genetic etiology, therefore genetic testing, such as targeted gene panels or whole exome/genome sequencing, is required to confirm the diagnosis and define the specific subtype of CDG (1). As next-generation sequencing technologies have become more accessible and affordable, they are increasingly used as first-line approach, allowing early identification of specific CDG. Early diagnosis is essential to start treatment promptly and try and improve clinical outcome (1, 4).

The clinical spectrum of PMM2-CDG is broad and heterogeneous, including neurological, hepatic, gastrointestinal, hematological and endocrine involvement (1, 5–7). Endocrinopathies, although frequently underrecognized, are being increasingly reported in patients with PMM2-CDG and may represent early or even presenting signs.

Glycoproteins are implicated in every endocrine axis (see **Table 1**), hence endocrinopathies are likely to occur in PMM2-CDG patients. The most frequent endocrine manifestations include growth failure (affecting approximately 50% of patients) and pubertal delay with hypergonadotropic hypogonadism reported in nearly all females (1, 8, 54). Thyroid function abnormalities are present in up to 75% of patients, often manifesting as transient hyperthyrotropinemia (35). Hyperinsulinemic hypoglycemia has been documented in 3-5% of cases, typically during infancy (40, 41). Lipid abnormalities, in particular hypobetalipoproteinemia, are observed in around half of patients (51). Although considered rare, recent studies suggest a higher prevalence of adrenal insufficiency (46).

**Table 1:** The impact of hypoglycosylation in several hormonal pathways, *in vitro* and *in vivo*.

Hormonal pathway	Known glycoproteins	*In vitro* effects of hypoglycosylation	Reported laboratory abnormalities	Reported clinical manifestations	References
Growth	ALS, IGFBP3, proIGF1Ea, IGF1R, GHR, GHBP, GHRHR	Reduced IGF1 secretion and half-life Reduced expression of IGF1R on the cell surface	Low IGF1, IGFBP3 and ALS; normal GH	Growth failure and final short stature	(1, 6–16)
Gonads	FSH, LH, FSHR, LHR, GnRHR	Reduced activation of FSHR by FSH Reduced expression of FSHR on the cell surface Reduced expression of LHR on the cell surface Reduced expression of GnRHR on the cell surface	Females: hypergonadotropic hypogonadism with elevated FSH and LH, low estrogen levels; low AMH Males: elevated FSH, low to normal testosterone	Females: POI, amenorrhea, delayed, incomplete or absent puberty, lack of secondary sexual characteristics Males: small testicular volume, cryptorchidism	(1, 4, 6, 8, 9, 17–34)
Thyroid	TSH, TSHR, TBG, Tg, TPO, TRHR	TBG reduced half-life Abnormal TSH-TSHR binding and signal transduction	Low TBG; elevated TSH (transitory); low fT4 (transitory); normal T3	Hypothyroidism (transitory)	(1, 6, 8, 9, 15, 19, 35–39)
Glucose metabolism	SUR1, insulin receptor	Reduced expression of KATP complex on the cell surface	Hypoglycemia, hyperinsulinism	Hypoglycemic events	(1, 40–45)
Adrenal gland	CRHR1, PC1/3, MC2R, CBG	Decreased ACTH secretion Reduced MC2R activity Reduced total circulating cortisol	Low ACTH and cortisol	Central adrenal insufficiency	(1, 46–50)
Lipid metabolism	INSIG1, PCSK9	Upregulation and decreased degradation of LDLR Decreased clearance of apoB-containing lipoprotein	Primary HBL: low TC, LDLc and apoB	N/A	(1, 51–53)

[ALS, acid-labil subunit; IGFBP3, Insulin-like growth factor-binding protein 3; GHR, growth hormone receptor; GHBP, growth hormone binding protein; IGF1R, insulin-like growth factor-1 receptor; IGF1, insulin-like growth factor-1; GH, growth hormone; GHRHR, growth hormone releasing hormone receptor; LH, luteinizing hormone; FSH, follicle stimulating hormone; FSHR, follicle stimulating hormone receptor; LHR, luteinizing hormone receotir; AMH, anti-mullerian hormone; POI, premature ovarian insufficiency; TSH, thyrotropin; TSHR, thyrotropin receptor; TBG, thyroxine-binding globulin; Tg, thyroglobulin; TPO, thyroperoxidase; TRHR, thyrotropin-releasing hormone receptor; fT4, free thyroxine; T3, triiodothyronine; SUR1, sulfonylurea 1 receptor; KATP, ATP-dependent potassium-channels; CRHR1, corticotropin-releasing hormone receptor 1; PC1/3, prohormone convertase 1/3; MC2R, melanocortin 2 receptor; CBG, corticosteroid-binding globulin; ACTH, adrenocorticotropin hormone; INSIG1, insulin-induced gene 1; SCAP, SREBP cleavage-activating protein; SREBP2, sterol regulatory element-binding protein 2; LDLc, low-density lipoprotein cholesterol; LDLR, low-density lipoprotein cholesterol receptor; PCSK9, protein convertase subtilisin/kexin Type 9; apoB, apolipoprotein B; TC, total cholesterol].

The endocrinological features of PMM2-CDG were reviewed in the past by Jaeken et al. in 1995 (54) and Miller et al. in 2003 (8). In this review we explore the endocrine system in PMM2-CDG in light of recent discoveries regarding the role of glycosylation on different hormonal pathways, with the purpose of prompting discussion on the management of endocrinological dysfunctions in these patients.

## Growth and GH-IGF1 axis

In the 2019 guidelines drawn up by Altassan and collaborators, it was estimated that half of all patients with PMM2-CDG exhibit short stature, typically associated to low levels of insulin-like growth factor 1 (IGF1), IGF binding protein 3 (IGFBP3) and acid-labile subunit (ALS) (1). Length, weight, and head circumference tend to be normal at birth, but a significant postnatal growth decline is commonly observed (7, 55, 56). In most cases, short stature is mild, slightly below -2 standard deviations (SD) and with sustained growth velocity. However, severe short stature has been reported, with height SD as low as -5.45 SD (7, 9, 55, 56).

**Figure 1:**
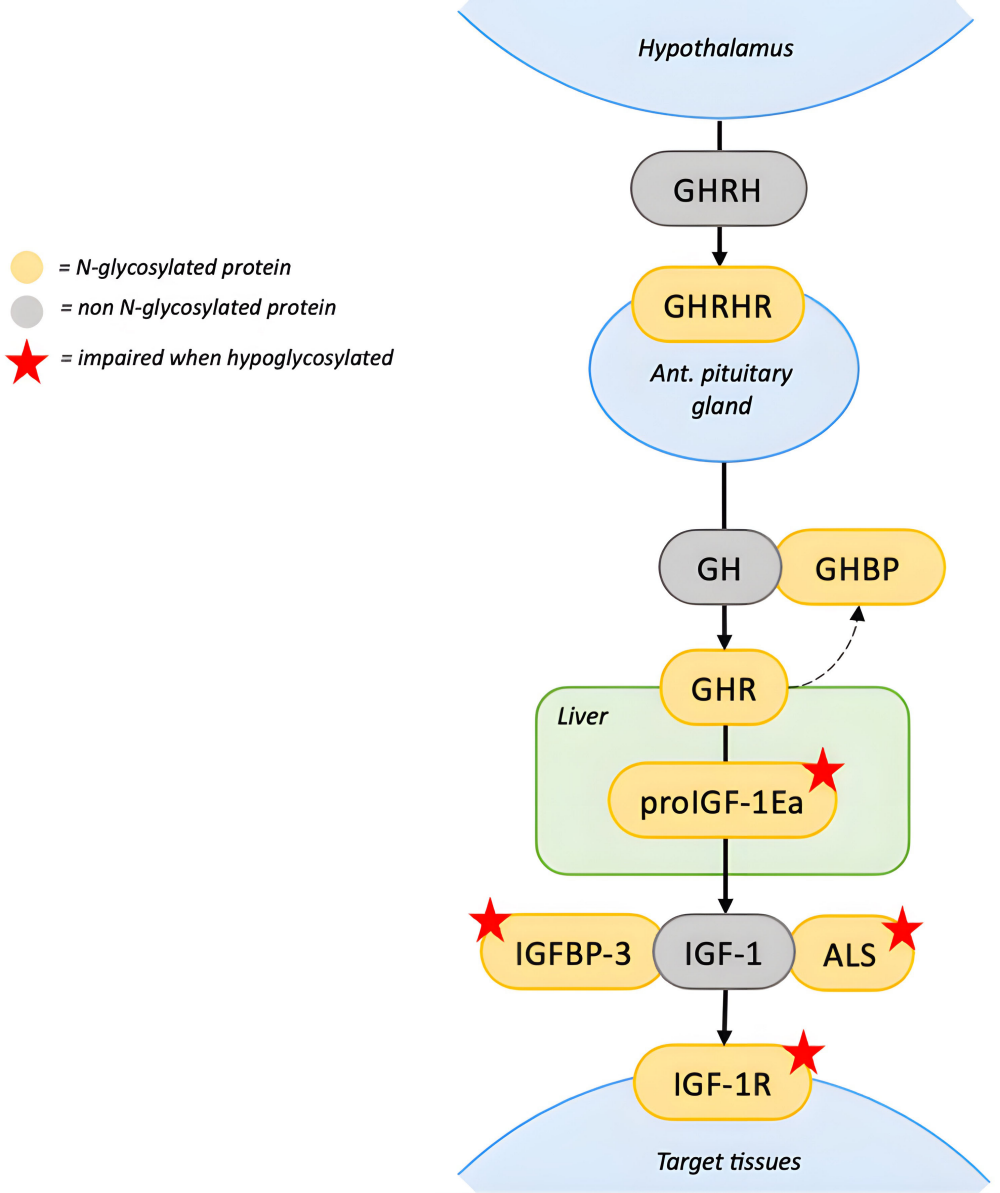
N-glycosylation in the GH-IGF1 axis. The GHRH, produced by the hypothalamus, stimulates its glycosylated receptor, GHRHR, on the anterior pituitary gland to release GH. GH binds to GHR on target tissues, including the liver. GHR contains N-linked glycosylation sites in the extracellular domain, which can be cleaved functioning as GHBP. Impact of hypoglycosylation on GHR and GHBP is unknown. In the hepatocytes, proIGF1Ea is the intracellular prohormone of IGF1. When the Ea domain of the prohormone is hypoglycosylated, mature IGF1 secretion is significantly reduced. In the circulation, IGFBP3 and ALS bind circulating IGF1 in a ternary complex. Hypoglycosylation destabilizes the complex reducing the half-life of circulating IGF1. On target tissues, IGF1 binds to IGF1R, which requires N-glycosylation to properly mature and translocate to the cell surface. Red stars indicate proteins whose function is impaired when hypoglycosylated. [ALS, acid-labil subunit; IGFBP3, Insulin-like growth factor-binding protein 3; GHR, growth hormone receptor; GHRH, growth hormone releasing hormone; GHRHR, growth hormone releasing hormone receptor; GHBP, growth hormone binding protein; IGF1R, insulin-like growth factor-1 receptor; IGF1, insulin-like growth factor-1; GH, growth hormone].

The nature of the growth impairment in these patients is likely to be multifactorial, involving nutritional, hormonal and genetic factors. During the first months of life, failure to thrive is usually attributed to poor feeding, malabsorption, and enteropathy (1). Although nutritional status often improves later in life, catch-up growth is generally absent (7, 55, 56). Impairment of the growth hormone (GH)-IGF1 cascade has been proposed as a potential mechanism for growth failure (8) (**Figure 1**).

The IGF1 pathway, essential for growth regulation, is particularly sensitive to hypoglycosylation. Serum IGF1 circulates in a ternary complex with the glycoproteins IGFBP3 and ALS, which stabilize it and prolong its half-life. Hypoglycosylation compromises the stability of this complex, thereby reducing the half-life of circulating IGF1 (10, 11). Mature IGF1 derives from post-translational cleavage of the heavily N-glycosylated C-terminal portion of the IGF1Ea prohormone (proIGF1Ea), named the Ea domain (11). When proIGF1Ea is hypoglycosylated, mature IGF1 secretion is significantly reduced (12). As a result, PMM2-CDG patients might have partial GH insensitivity, with normal GH production but low levels of IGF1.

Moreover, the IGF1 receptor (IGF1R) requires N-glycosylation to properly mature and translocate to the cell surface. PMM2-CDG fibroblast showed an impairment of IGF1 signaling pathway transduction, suggesting a degree of IGF1 resistance (11). Patients with congenital IGF1 or IGF1R defects show growth failure associated with microcephaly, heart defects, and dysmorphic features, similar to clinical features observed in PMM2-CDG patients (57). However, complete IGF1 resistance is associate to intrauterine growth restriction, which is not observed in PMM2-CDG, indicating a partial defect (58).

Less is known about the effects of hypoglycosylation on other hormones of the GH-IGF1 axis. Growth hormone–releasing hormone (GHRH) binds to its glycosylated receptor (GHRHR) on the pituitary gland to stimulate GH production. Hypoglycosylation of GHRHR could potentially impair GH secretion, however, GH deficiency has not been reported in PMM2-CDG nor has GH stimulation testing been performed (59). GH itself is non-glycosylated while its receptor (GHR) contains five N-linked glycosylation sites in the extracellular domain (13). This domain can be cleaved from the GHR and circulate as GH binding protein (GHBP) (14). The role of GHBP is unclear and it is still unknown whether hypoglycosylation of GHR and GHBP compromises the GH signaling pathway.

A genotype-specific effect on growth patterns has also been suggested. PMM2-CDG patients heterozygous for the p.Arg141His variant, which is the most frequent mutation (estimate prevalence of 45-60%), often show a more pronounced growth impairment (7). It affects the catalytic site of PMM2, expressing no or very limited residual enzyme activity when the second allele is a severe variant (i.e. p.Phe119Leu mutation), resulting in early severe presentation with feeding problems, growth failure, hypotonia, and development delay (3, 15).

In early infancy, prevention of growth failure is mainly based on nutritional support (1). Otherwise, there is no consensus on the endocrinological management of short stature. During childhood and adolescence, anthropometric measurements should be obtained at every visit or at least every six months, alongside annual assessments of IGF1 and IGFBP3. Bone age should be evaluated at the onset of puberty (1, 60). Although not reported in the literature, dynamic testing (i.e. GH stimulation test and IGF1 generation test) should be considered in patients with short stature and low IGF1 and IGFBP3 levels, to identify those who may benefit from hormonal replacement therapy (recombinant human GH – rhGH – or IGF1 – rhIGF1). Notably, treatment with rhIGF1 restored linear growth in a child with PMM2-CDG and abnormal IGF1 generation test, suggesting that rhIGF1 treatment may improve the clinical outcome in these patients (11, 16).

## Puberty and gonadal axis

Pubertal abnormalities are frequently reported in PMM2-CDG, especially in females (1), and are primarily caused by an impairment of the hypothalamic-pituitary-gonadal axis. Their expression in both sexes is variable and although no clear genotype-phenotype correlation has been established, it is likely that mutations with greater impact on glycosylation could be associated with more severe pubertal abnormalities.

### Female patients

Hypergonadotropic hypogonadism is a hallmark feature in PMM2-CDG female patients (1). Most females exhibit elevated follicle stimulating hormone (FSH) and luteinizing hormone (LH) associated with low serum estradiol and anti-Müllerian hormone (AMH) (6, 9, 17). Pubertal development is typically delayed, incomplete, or entirely absent, though two females with normal puberty have been described (18, 19). Premature ovarian insufficiency (POI), amenorrhea and lack of secondary sexual characteristics are common (1). Ovarian agenesis has been found in some cases by laparoscopy and ultrasound examination (17, 20).

**Figure 2:**
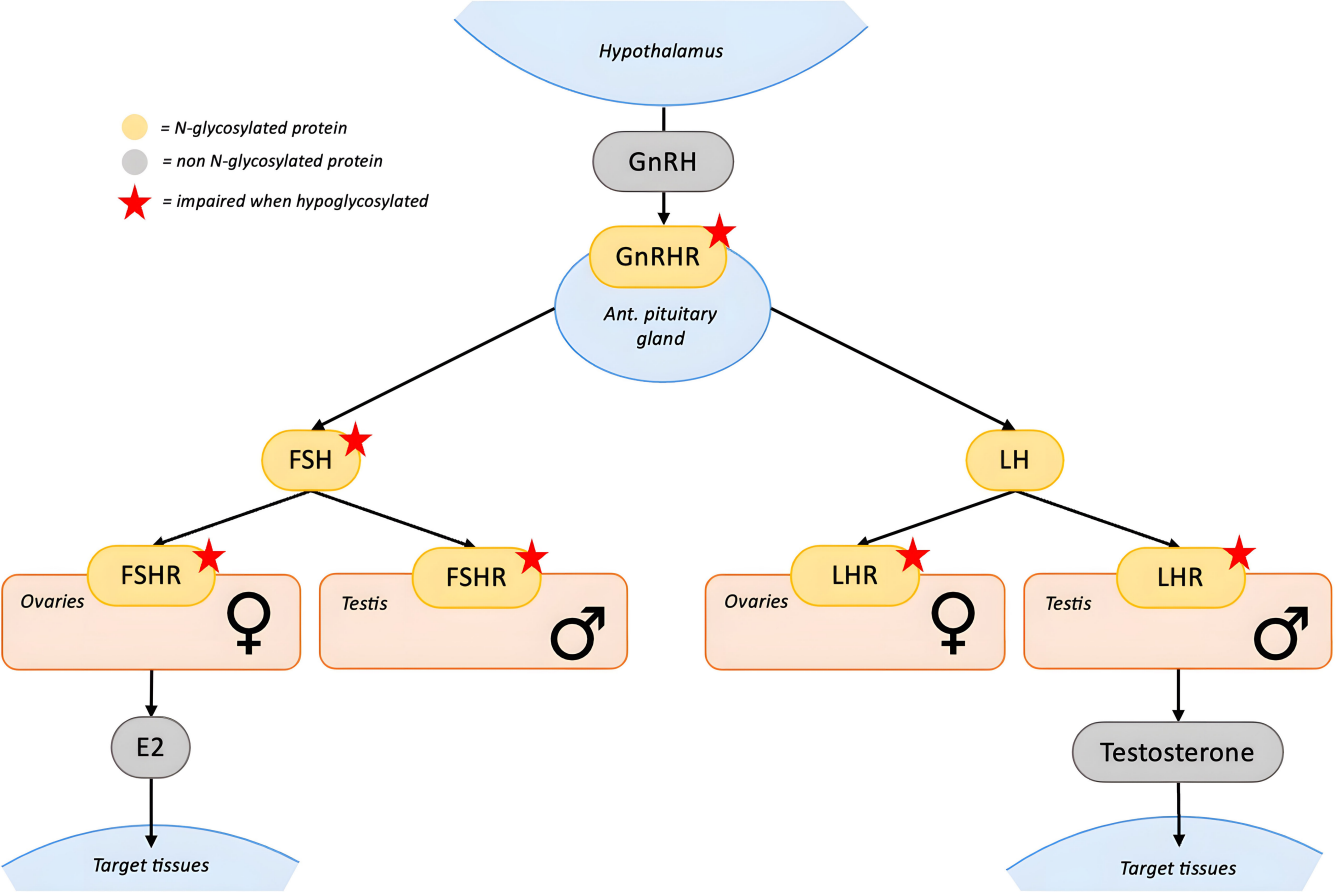
N-glycosylation in the Gonadal axis. GnRH is secreted by the hypothalamus and stimulates the anterior pituitary through GnRHR to release FSH and LH. These N-glycosylated gonadotropins act on their respective N-glycosylated receptors (FSHR and LHR) located in the gonads. In the ovaries, FSH promotes estradiol (E2) production and follicular development, while LH regulates ovulation and luteinization. In the testes, FSH acts on Sertoli cells to support spermatogenesis, whereas LH stimulates Leydig cells to produce testosterone. Glycosylation is essential for the proper folding, trafficking, and function of FSH, FSHR and LHR, while it has less impact on LH bioactivity. Red stars indicate proteins whose function is impaired when hypoglycosylated. [GnRH, gonadotropin-releasing hormone; FSH, follicle-stimulating hormone; LH, luteinizing hormone; FSHR, FSH receptor; LHR, LH receptor].

Hypoglycosylation of FSH and its receptor (FSHR) plays a central role in the pathogenesis of pubertal abnormalities (**Figure 2**). FSH is formed by an α- and a β-subunit, each containing two glycosylation sites (21). The degree of glycosylation is variable and functionally significant, since specific FSH glycoforms modulate estradiol secretion, follicular growth, and antral formation (21). Furthermore, proper FSH glycosylation is necessary for full activation of the FSHR (22, 23), potentially by enabling conformational changes within the FSH–FSHR complex or through interactions between oligosaccharides and the receptor’s transmembrane domain (21). FSHR itself contains multiple N-linked glycosylation sites that contribute to proper protein folding, stability and translocation to the plasma membrane. Hypoglycosylation could therefore impair the maturation of newly synthesized FSHR contributing to the FSH axis dysfunction (23).

The impact of hypoglycosylation on other hormones of the hypothalamic–pituitary–gonadal axis is less evident. Both LH and its receptor (LHR) are glycoproteins, and while hypoglycosylation does not appear to affect LH bioactivity and bioavailability, it may impair LHR maturation and reduce its surface expression (24–26). Gonadotropin-releasing hormone (GnRH) stimulates gonadotropin release via its glycosylated receptor (GnRHR). Hypoglycosylation may reduce GnRHR membrane expression (27), however its clinical relevance appears limited as most female patients exhibit elevated gonadotropin levels.

Pubertal abnormalities, especially POI, are not exclusively dependent on gonadotropin dysfunction (17). Studies from murine models and fetal human ovaries suggest that PMM2 is highly expressed in oocytes and plays an important role in early follicular development (28–30). Therefore, hypoglycosylation due to PMM2 deficiency may directly compromise oogenesis and early folliculogenesis causing POI in a gonadotropin-independent way (17), potentially explaining the low AMH levels observed in these patients. This distinguishes PMM2-CDG from other forms of hypogonadism primarily driven by gonadotropin dysfunction, such as FSHR mutations, where early follicular development remains intact (61, 62).

Pubertal development should be monitored at each visit using Tanner staging. At pubertal age, gonadotropin, 17-beta-estradiol, AMH and pelvic ultrasound should be performed. Although mini-puberty has not been described in PMM2-CDG, assessing gonadotropin levels during this phase may help identify patients at high risk of developing hypergonadotropic hypogonadism. PMM2-CDG females with hypergonadotropic hypogonadism benefit from low-dose estrogen therapy for puberty induction, long-term prevention of osteopenia and overall improvement of quality of life (1, 31). Beginning the treatment around 12 y/o and gradually increasing the dosage of estrogen during the first two to three years is recommended. Progestin should be added after two years, or earlier if uterine bleeding occurs. Transdermal administration is recommended, since oral estrogen replacement has been linked to thrombosis and CDG patients often exhibit coagulopathy. In selected cases prophylactic anticoagulation should also be considered (63). In adult patients, bone density monitoring is advised and calcium and vitamin D supplementation may be indicated (1).

### Male patients

Pubertal abnormalities are less common in PMM2-CDG male patients (1). Some patients exhibit delayed or incomplete puberty with small testicular volume, increased FSH and normal LH. Testosterone levels can be in the low-normal range, though reference ranges may not account for pubertal stage and testicular volume, potentially reflecting delayed development rather than reduced testosterone secretion (31–33). Cryptorchidism has been reported in PMM2-CDG but there is no evidence of association with hypogonadism later in life (8, 64).

At the molecular level, the effects of hypoglycosylation are similar to those described in females and primarily involve the FSH-FSHR cascade (34). In males, FSH promotes spermatogenesis and testicular growth, therefore, an impaired FSH-FSHR function could explain the reduced testicular volume observed in some patients. While spermatogenesis has not been studied in PMM2-CDG, impaired spermatogenesis is a known feature in patients with FSHR mutation, suggesting a possible similar dysfunction in PMM2-CDG (65). Most patients exhibit normal testosterone levels and normal development of secondary sexual characteristics suggesting a preserved LH function.

As with females, endocrine follow up should include monitoring of pubertal progression and assessment of hormonal levels, including gonadotropins and testosterone, also during mini-puberty. Testosterone replacement therapy can be considered in patients with delayed puberty and biochemical evidence of hypogonadism, however, a consensus statement on the management of pubertal induction in PMM2-CDG males in currently lacking.

## Thyroid axis

**Figure 3:**
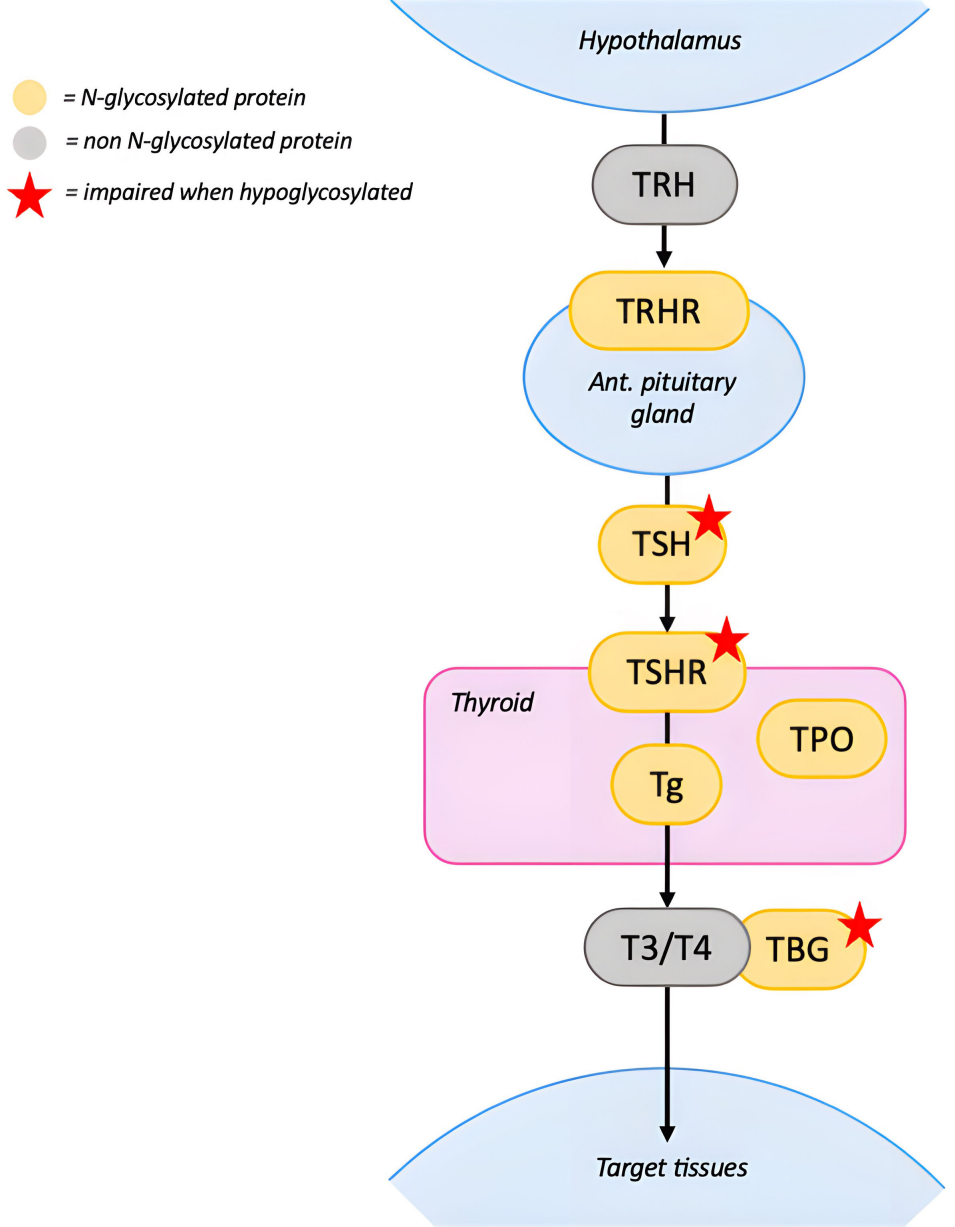
N-glycosylation in the Hypothalamus-Pituitary-Thyroid axis. TRH stimulates the pituitary gland to release TSH, which binds to TSHR in the thyroid. Hypoglycosylation of TSH and TSHR impairs binding and signal transduction. The thyroid produces T3 and T4 from Tg, this process requires multiple enzymes, including TPO. Impact of hypoglycosylation on Tg and TPO is unknown. Once released in the circulation T3 and T4 bind to TBG to reach target tissues. Hypoglycosylation of TBG reduces its half-life but doesn’t seem to affect its ability to bind thyroid hormones. Red stars indicate proteins whose function is impaired when hypoglycosylated. [TRH, thyrotropin releasing hormone; TSH, thyrotropin; TSHR, thyrotropin receptor; TBG, thyroxine-binding globulin; Tg, thyroglobulin; TPO, thyroperoxidase; T4, thyroxine; T3, triiodothyronine].

Most of the proteins implicated in the thyroid axis are N-glycosylated, including thyroxine-binding globulin (TBG), thyrotropin (TSH), TSH receptor (TSHR), thyroglobulin (Tg), thyroperoxidase (TPO) and thyrotropin-releasing hormone receptor (TRHR) (**Figure 3**). Three quarters of PMM2-CDG patients exhibit laboratory abnormalities related to the hypothalamus-pituitary-thyroid axis (1).

Partial TBG deficiency is the most frequently reported (1). Hypoglycosylation reduces TBG half-life and stability without significantly affecting its ability to bind thyroid hormones (1, 8, 36). Although partial TBG deficiency does not require treatment, it may serve as an early laboratory marker for CDG in infancy (37).

Hyperthyrotropinemia is another common finding in PMM2-CDG (6, 9, 19, 35, 38). TSH contains three N-glycosylation sites: two on the α-subunit, which affect signal transduction after binding to TSHR, and one on the β-subunit, which is important for TSH stability and secretion (39). Hypoglycosylation could reduce TSH bioactivity leading to compensatory increases in TSH. Moreover, TSHR requires proper glycosylation for effective ligand binding, therefore its impairment may further contribute to the hyperthyrotropinemia (39).

Hyperthyrotropinemia is generally transitory and rarely associated with low levels of free thyroxin (fT_4_) and triiodothyronine (T_3_) (8), although up to 15% of patients require temporary treatment with Levothyroxine (LT_4_) (1). Clinical signs of hypothyroidism (such as myxedema, weight gain, and constipation) are not frequently reported, probably because they overlap with PMM2-CDG clinical manifestations (35). Transient TSH increase is more common during the first months of life and may be detected at neonatal screening (35). During this phase, thyroid hormone requirement and production are physiologically increased, suggesting that the effects of hypoglycosylation on thyroid function may become clinically manifest under conditions of increased demand.

It would be interesting to evaluate whether there is a genotype-phenotype correlation with thyroid dysfunction among CDG patients. It is plausible that, the more glycosylation is compromised, the greater the impact on thyroid function.

There are no reports on thyroid autoimmune disorders in PMM2-CDG patients, probably because autoantibodies are rarely considered in these patients. Interestingly, major thyroid antigens (Tg, TSHR and TPO) are glycosylated and a lower grade of glycosylation seems to reduce their immunogenicity (35).

Thyroid function screening ought to be carried out for all PMM2-CDG patients diagnosed *de novo* and repeated yearly during follow up. When hyperthyrotropinemia is present, LT_4_ replacement therapy is indicated if fT_4_ is low or if there are clinical signs of hypothyroidism. It is important to exclude isolated hyperthyrotropinemia before starting LT_4_ treatment. During treatment, TSH and fT4 levels should be regularly monitored, and therapy should be reduced or discontinued if TSH becomes suppressed or fT4 levels are elevated, since hypothyroidism is commonly transient (38).

## Glucose metabolism and the insulin axis

Hypoglycemia is less prevalent in PMM2-CDG than in other CDG subtypes, occurring in approximately 3-5% of patients (40, 41). When present, it typically occurs during early infancy and may represent an early diagnostic clue. Clinical presentation ranges from asymptomatic hypoglycemia discovered during routine testing to rare but severe events such as decreased consciousness, hypotonia, and seizures (40, 41).

No genotype-phenotype correlation has been established for hypoglycemia. However, the most common p.(Arg141His) allele is underrepresented in patients with hypoglycemic events (30% versus 60% in all the PMM2-CDG reported cases) (40).

The pathogenesis of hypoglycemia in PMM2-CDG patients is unclear. Hypoglycemic events occurring during the neonatal period and early infancy are likely caused by poor feeding and failure to thrive. Hyperinsulinism is another important cause of hypoglycemia and has been confirmed in nearly half of the reported cases (40, 41).

**Figure 4:**
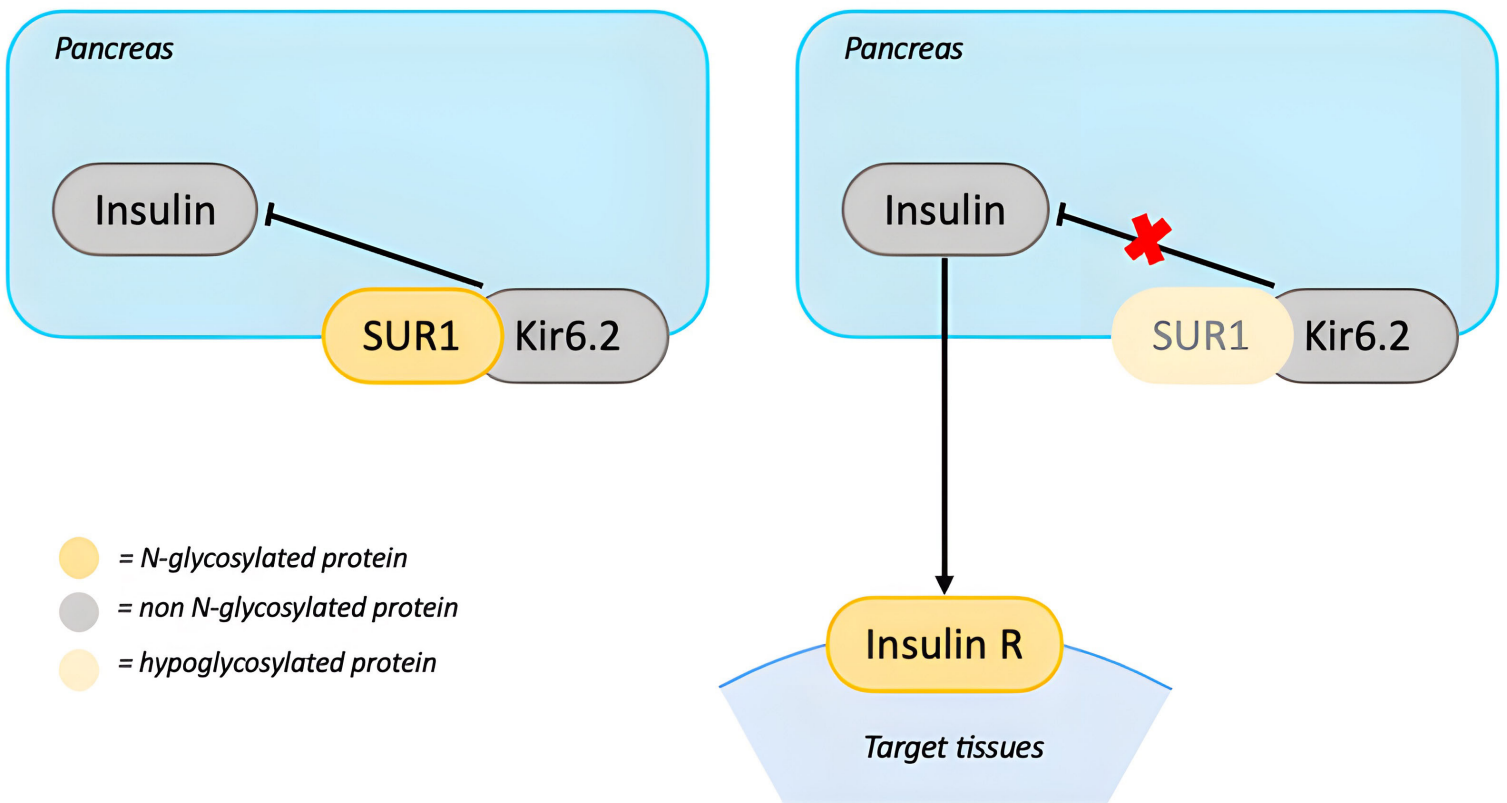
N-glycosylation in the Insulin axis. In presence of euglycemia, excess of insulin release is prevented by beta-cell depolarization, which is obtained through the ATP-dependent potassium-channels (KATP). KATP is a complex which includes SUR1 and Kir6.2. Hypoglycosylation of SUR1 could impair KATP’s function leading to excessive insulin release and hypoglycemia. On target cells insulin binds to Insulin R. The impact of hypoglycosylation of this receptor is still unknown. [SUR1, sulfonylurea 1 receptor; Insulin R, insulin receptor].

PMM2-CDG patients with hyperinsulinemic hypoglycemia generally respond well to treatment with diazoxide. Diazoxide opens ATP-dependent potassium-channels (KATP) on the beta cells of the pancreas, preventing cellular depolarization and inhibiting insulin secretion (42). The KATP channel is a functional complex which includes the sulfonylurea 1 receptor (SUR1) and Kir6.2, an inward rectifier potassium channel subunit. SUR1 requires glycosylation for stabilization and translocation of KATP on the cell surface. Hypoglycosylation due to PMM2 deficiency could impair the function of the KATP complex leading to increased insulin secretion and hypoglycemia (42) (**Figure 4**).

Another potential mechanism underlying hyperinsulinism is an impairment of the insulin receptor (INSR). Proper N-glycosylation of INSR is essential for its processing and signal transduction (43, 66–68). It has been hypothesized that INSR hypoglycosylation during the fetal period may trigger compensatory hyperinsulinemia *in utero*, potentially persisting after birth and contributing to neonatal hyperinsulinemic hypoglycemia (69). Nevertheless, insulin resistance has not been described in PMM2-CDG, thus the postnatal effect of INSR hypoglycosylation remains uncertain.

Muller et al. in 2004 (70) and Cabezas et al. in 2017 (71) identified a pathogenetic variant in the *PMM2* gene promoter which reduces gene transcription selectively in pancreas, liver and kidney. This phenotypic variant of PMM2-CDG is characterized by polycystic kidney, liver disease and hyperinsulinemic hypoglycemia responsive to diazoxide (72). This strengthens the hypothesis that hypoglycosylation caused by PMM2 deficiency can affect insulin function.

As hypoglycemic events can be the presenting sign of the disease, PMM2-CDG should be included in the differential diagnosis of persistent hypoglycemia in newborns or infants (44, 73).

Hypoglycemic events should never be underestimated in PMM2-CDG. Blood sugar should be monitored in these patients mainly in the first years of age and when feeding difficulties are present. In the event of hypoglycemia, before starting glucose infusion a blood sample should be promptly collected in order to measure plasma insulin, cortisol, GH, lactic acid, ammonia, beta-hydroxybutyrate, free fatty acids and urinary ketones (1). If hyperinsulinism is present, a trial with diazoxide is advised. Although generally safe, daizoxide requires careful monitoring for potential side effects. In one patient diazoxide was suspended due to refractory hyponatremia, subsequent subtotal pancreatectomy prompted good glycemic control (45). In about one third of the patients hyperinsulinism may be transient, and since the risk of hypoglycemic events lowers later in life, tapering and discontinuation of diazoxide can be considered, especially once feeding difficulties improve.

## Adrenal axis

In PMM2-CDG adrenal function has always been considered normal, hence measuring serum cortisol and ACTH is not currently part of recommended routine screening (1). Probably for this reason, adrenal function has rarely been the object of reports in the literature. However, in CDGs in general, cortisol levels tend to be low to normal (64). In 2021, an observational study by Čechová et al. (46) indicated that adrenal insufficiency (AI) may be part of the PMM2-CDG phenotype and may therefore be underestimated. In a cohort of 43 patients, 25% had low morning cortisol levels with normal to low ACTH and 5% had confirmed central adrenal insufficiency (CAI) following ACTH stimulation test (46). No patients with primary AI were reported.

**Figure 5:**
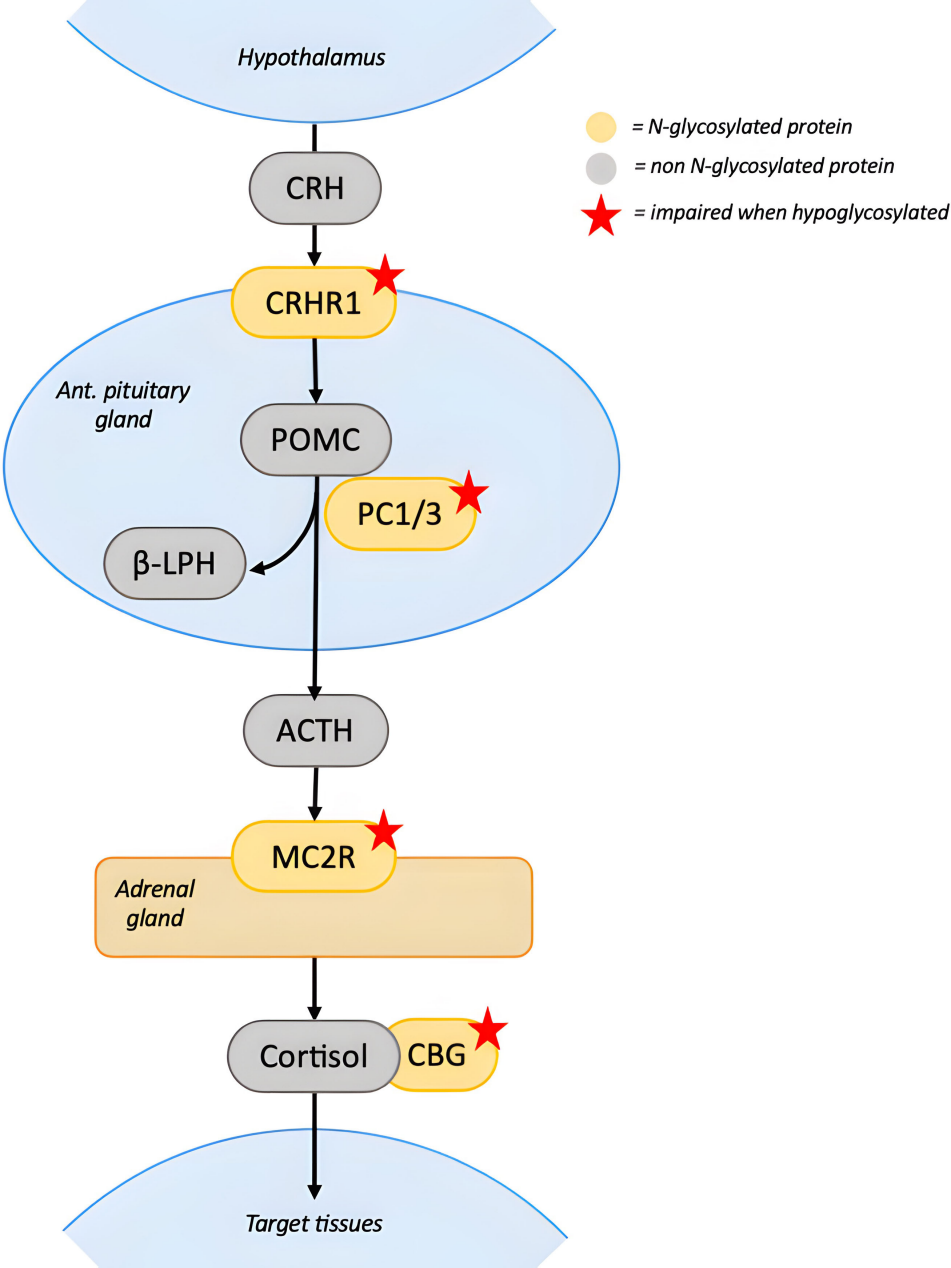
N-glycosylation in the Hypothalamus-Pituitary-Adrenal axis. CRH activates CRHR1 on the pituitary gland to stimulate ACTH’s production and secretion. Hypoglycosylation of CRHR1 impairs binding to CRH and signal transduction. In the pituitary, POMC is cleaved by PC1/3 to release BLPH and ACTH. PC1/3 activity could be impaired by hypoglycosylation, leading to decreased ACTH secretion. ACTH stimulates cortisol’s secretion on the adrenal gland by binding MC2R and the activation of the receptor depends on its glycosylation state. In the circulation, cortisol binds to CBG. Dysfunction of CBG due to hypoglycosylation decreases circulating cortisol levels. Red stars indicate proteins whose function is impaired when hypoglycosylated. [CRH, corticotropin-releasing hormone; CRHR1, corticotropin-releasing hormone receptor 1; PC1/3, prohormone convertase 1/3; POMC, proopiomelanocortin; beta-lipotropic hormone; ACTH, adrenocorticotropin hormone; MC2R, melanocortin 2 receptor; CBG, corticosteroid-binding globulin].

As for the other endocrine axes, many of the proteins involved in the adrenal pathway are N-glycosylated (46) (**Figure 5**). Physiologically, ACTH secretion is induced by CRH receptor 1 (CRHR1) activation mediated by corticotropin-releasing hormone (CRH). Hypoglycosylation of CRHR1 impairs binding to CRH and signal transduction, preventing ACTH release (47). In the pituitary, prohormone convertase 1/3 (PC1/3) cleaves preproopiomelanocortin to produce beta-lipotropic hormone (BLPH) and ACTH. Since PC1/3 is glycosylated, hypoglycosylation could also impair BLPH and ACTH production (48). Additionally, the melanocortin 2 receptor (MC2R, ACTH receptor) in the adrenal gland and the corticosteroid-binding globulin (CBG) in the circulation both rely on glycosylation for proper function (49, 50). The combined impairment these glycoproteins could lower cortisol synthesis and half-life.

Clinical consequences of AI, such as adrenal crises, can be life-threatening, hence more attention should be paid to the evaluation of AI in PMM2-CDG patients. As proposed by Čechová et al. (46), adrenal function should be screened at least yearly by measuring morning cortisol and ACTH. In patients with low cortisol levels, low-dose ACTH stimulation test should be performed to confirm the diagnosis of CAI. In case of hypoglycemia or other symptoms indicative of an adrenal crisis, cortisol levels should be assessed. In patients with confirmed CAI, replacement therapy with hydrocortisone should be started at physiological dose (8-10 mg/m^2^ daily divided in two to three doses) and each patient should be educated on stress dosing and on the use of emergency steroid kits.

Further studies are needed to determine the long-term outcomes of PMM2-CDG patients with CAI, especially to determine whether adrenal function varies with age. Moreover, targeted studies are needed to establish whether there is a correlation between genotype and risk of AI.

## Lipid metabolism

Approximately half of PMM2-CDG patients exhibit abnormalities in lipid metabolism (6, 9, 38), most commonly primary hypobetalipoproteinemia (HBL), which is characterized by low plasma levels of total cholesterol (TC), low-density lipoprotein cholesterol (LDLc) and apolipoprotein B (apoB) (51).

**Figure 6:**
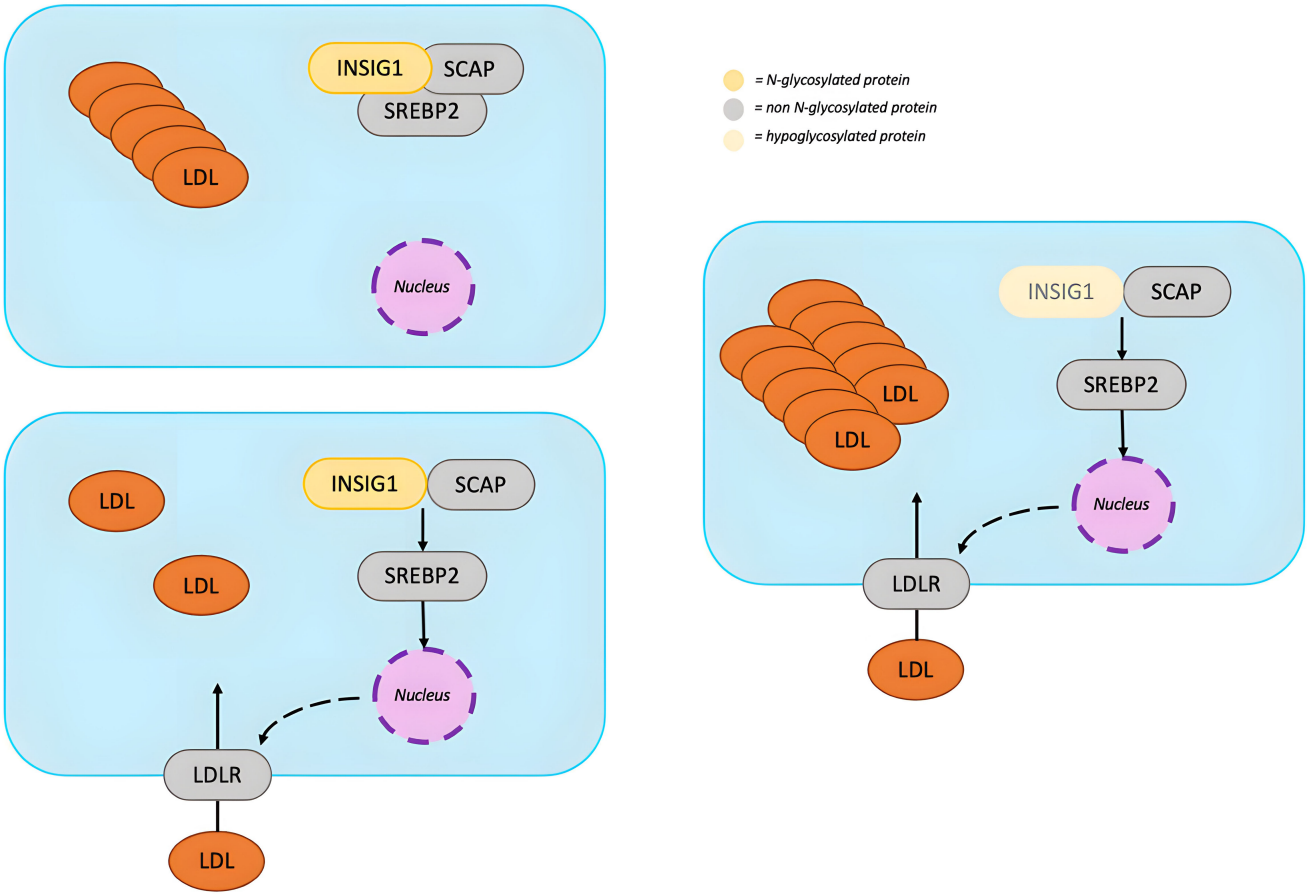
N-glycosylation in lipid metabolism. When intracellular concentration of sterols decreases, SREBP2 is released from the INSIG1-SCAP complex and, after proteolytic activation, translocates into the nucleus where it induces the transcription of genes involved in cholesterol synthesis, including the LDLR gene. LDLR removes apoB-containing lipoproteins from circulation until the intracellular concentration of sterols is restored. Hypoglycosylation of INSIG1 could impair its binding to SREBP2, leading to an upregulation of LDLR and increased clearance of apoB-containing lipoproteins. [INSIG1, insulin-induced gene 1; SCAP, SREBP cleavage-activating protein; SREBP2, sterol regulatory element-binding protein 2; LDL, low-density lipoprotein; LDLR, low-density lipoprotein cholesterol receptor].

Occurrence of HBL in PMM2-CDG seems to be prompted by an increased clearance of apoB-containing lipoproteins which is mediated by an increase in Low Density Lipoprotein Receptor (*LDLR*) gene expression (51) (**Figure 6**). *LDLR* transcription is induced by sterol regulatory element-binding protein 2 (SREBP2). When the intracellular concentration of sterols is high, SREBP2 is retained in the endoplasmic reticulum (ER) by binding SREBP cleavage-activating protein (SCAP) and insulin-induced gene 1 (INSIG1). When intracellular concentration of sterols decreases, SREBP2 undergoes proteolytic activation and induces the transcription of genes involved in cholesterol synthesis, including the *LDLR* gene. Abnormal N-glycosylation probably impairs INSIG1 preventing SREBP2 retention in the ER (51). An excessive SREBP2 activation upregulates LDLR promoting removal of apoB-containing lipoproteins from circulation. Another contributor could be a dysfunction of the protein convertase subtilisin/kexin Type 9 (PCSK9), which can bind LDLR activating its degradation (52). Although hypoglycosylation does not seem to alter PCSK9 folding and secretion, it may reduce its ability to enhance LDLR degradation (53). Some studies report lower PCSK9 levels in patients with PMM2-CDG compared with healthy controls (52).

Given the short life expectancy of PMM2-CDG patients, the clinical implications of HBL are unknown, particularly whether low LDLc levels could be protective against atherosclerosis. Lipid profile (total cholesterol, LDLc, HDLc, triglycerides, apoB) should be obtained at the time of PMM2-CDG diagnosis and monitored every 1–2 years, especially in patients with poor nutritional status (1). Currently, there is no indication for lipid supplementation in this population.

Future research should explore whether lipid abnormalities could serve as early biomarkers for PMM2-CDG or if they correlate with specific genotypes. Furthermore, studies on glycosylated regulators such as PCSK9 and INSIG1 may provide insights into cholesterol metabolism regulation and identify potential therapeutic targets for other lipid metabolism disorders.

## Future perspectives

PMM2-CDG is a complex disorder that requires multidisciplinary care. The inclusion of endocrinologists in the multidisciplinary team is essential to ensure early identification and appropriate management of hormonal dysfunction. Moreover, this may encourage the development of consensus guidelines to standardize protocols for screening, diagnosis and treatment of endocrine abnormalities, especially for high-priority clinical concerns such as pubertal delay.

The wide range of clinical phenotypes highlights the need for personalized care in order to improve clinical outcomes and quality of life. Although genotype-phenotype correlations have been explored, limited data are available linking specific genotypes to endocrine abnormalities (74). The high frequency of compound heterozygosity makes these associations more difficult to establish. Large international registries will be useful to identify correlations and to develop genotype-specific monitoring strategies.

Unfortunately, at present there are no available disease-modifying or curative treatments for PMM2-CDG, and management relies mainly on symptomatic and support treatments (1, 4, 75). There have been multiple attempts to identify a curative treatment for PMM2-CDG, most of which focus on the substrate replacement therapy including mannose, mannose-1-phosphate (alone or in liposomes) or its derivatives with increased lipophilicity (75–78). Other therapeutic approaches, such as pharmacological chaperons and aldose reductase inhibitors, are still pre-clinical or at an early stage of clinical testing (75, 79–81). Furthermore, to date, there is no literature focused on the impact of these treatments on the endocrine system in PMM2-CDG. Monitoring of hormonal function in future clinical trials could evaluate whether these novel therapies could be beneficial also on endocrine abnormalities. We can speculate that a treatment effective in restoring the N-glycosylation assembly chain would improve the overall endocrine function. If this is the case, certain endocrine abnormalities, such as TBG deficiency or HBL, could potentially serve as biomarkers of treatment efficacy. The therapeutic benefit is likely to be more clinically significant if started early, especially to restore linear growth. However, certain abnormalities, such as impairment of early folliculogenesis leading to POI in females, may remain uncorrected.

## Conclusion

The number of patients receiving a diagnosis of CDG is increasing, making it important to understand the endocrine function thoroughly in these conditions and investigate whether treatment of related endocrine disorders can be improved. We reviewed the potential role of glycosylation on hormonal pathways to summarize the main endocrinological features of PMM2-CDG. *In vitro* studies have shown that glycoproteins play a role along various hormonal axes, acting not only as hormones but also as precursor hormones, receptors, enzymes, and transport proteins. Clinical studies on affected patients provided insights into the real-life importance of glycosylation on the proper functioning of the endocrine system. The key biochemical and clinical findings across the different axes are summarized in **Table 1**. Based on these findings, we proposed practical recommendations for clinical assessment and management, summarized in **Table 2**.

**Table 2:** Proposed approach to screening, diagnosis, and treatment of endocrine abnormalities in PMM2-CDG.

Endocrine Axis	Endocrine dysfunction	Screening	Diagnosis	Treatment options
Growth	Growth failure	Height, BMI and growth velocity assessment every 6 months Monitor IGF1, IGFBP3 yearly Assess bone age at puberty	Short stature, low IGF1 and IGFBP3 Consider dynamic tests (GH stimulation test and IGF1 generation test) to document GH deficiency or insensitivity	Improve nutritional status If GH deficiency is present consider rhGH If GH insensitivity is present consider rhIGF1
Gonads	Delayed or incomplete puberty	Tanner staging every 6 months At mini-puberty and puberty: *In females*: Monitor FSH, LH, estradiol and AMH yearly; Baseline pelvic ultrasound *In males*: Monitor FSH, LH and testosterone yearly	Absence or lack of progression of clinical signs of puberty and: *In females*: high LH and FSH, low estradiol and low AMH *In males*: high FSH, normal LH, normal to low testosterone	*In females*: Transdermal estrogens and oral progestin. Consider concurrent antithrombotic prophylaxis. *In males*: Consider intramuscolar testosterone
Thyroid	Hypothyroidism	Monitor TSH, fT4, T3 yearly Investigate signs and symptoms of hypothyroidism	Elevated TSH with normal or low fT4	Levothyroxine if fT4 is low or symptoms are present.
Glucose metabolism	Hypoglycemia and hyperinsulinism	Blood glucose yearly When symptomatic: blood glucose, insulin, GH, cortisol, ketones, free fatty acids, ammonia	Hypoglycemia with elevated insulin	Diazoxide Consider partial pancreatectomy in selective refractory cases
Adrenal gland	Central adrenal insufficiency	Morning cortisol and ACTH yearly Assess cortisol if hypoglycemia or other symptoms suggestive of adrenal insufficiency	Low cortisol with normal/low ACTH, to be confirmed by ACTH test	Hydrocortisone
Lipid metabolism	Primary hypo-betalipoproteinemia	Total cholesterol, LDLc, HDLc, apoB every 1–2 years	Low LDLc and apoB	None indicated

[GH, growth hormone; IGF1, insulin-like growth factor 1; IGFBP3, insulin-like growth factor-binding protein 3; FSH, follicle-stimulating hormone; LH, luteinizing hormone; AMH, anti-Müllerian hormone; TSH, thyroid-stimulating hormone; fT4, free thyroxine; T3, triiodothyronine; LDLc, low-density lipoprotein cholesterol; HDLc, high-density lipoprotein cholesterol; apoB, apolipoprotein B; ACTH, adrenocorticotropic hormone; HBL, hypobetalipoproteinemia; rhGH, recombinant human growth hormone; rhIGF1, recombinant human insulin-like growth factor 1].

As the biochemical effects of hypoglycosylation on different endocrinological axes become increasingly clear, further clinical research involving larger patient cohorts is needed to establish consensus guidelines for the management and treatment of endocrinopathies in PMM2-CDG.
